# A Digest

**Published:** 1895-08

**Authors:** Carl Fischer

**Affiliations:** Zanesville, O.


ARTICLE VI.

### A DIGEST.

BY CARL FISCHER, D. D. S., ZANESVILLE, O.

In following dental literature the *pro* and *con* arguments about subjects of vital interest to dentists make it sometimes difficult to arrive at a definite conclusion Following the teachings of authority blindly is not altogether satisfactory to the intelligent dentist, and experiments should always go hand in hand, to establish the truth of an investigation in one's own mind. When sulphuric acid came to the front as an aid to enlarge root canals, I made solutions from 30 to 50 per cent. of strength and found their action on tooth structure very slow. Calcium phosphate is scarcely attacked by the cold acid and of calcium carbonate we have very little. The acid to dissolve a tooth is nitric acid (of course to be used with judgment). The best way to use it is probably to add to a 50 per cent. solution of sulphuric acid enough to increase action, say 10 per cent. Dilute nitric acid will dissolve steel, while concentrated will not, therefore a steel broach cannot be used. The action of nitric acid varies with the concentration, 3 drops in water is recommended for stomatitis (Hare), while the concentrated form is a powerful escharotic. Where cauterization of the apical space is desirable, surely no objection can be brought forward. Dr. Barrett uses the strong acid in case of necrosis. To stop the action of nitric acid on tissue, soap is the best agent to employ. (Soap can best be dissolved in dilute alcohol )

Have you used Iodine and Cassia? (Dental Digest No. 2.) Does this mean alone or combined? or for what purpose? or is it a specific? Well both are irritants in a root-canal as far as my observations go, and help to break down tissue.

In reading Dr. Dudley's article on "Titles," no one can but feel the truth expressed in this timely article. I think with Dr. Mears (and if any one knows, he does) that a good dental college furnishes all the mental food necessary to become an excellent dentist. To do justice to both studies in four years means a fine education beforehand.

Ethics now and then have an airing in the dental journals, but it does not influence anybody in the slightest degree. Following the modern principles, honor becomes a back number. Not mentioning the common everyday advertising dentist, but turning our attention to the college professor, we find a special code of ethics for him. He does not think anything of blowing his horn till everyone becomes nauseated. So that his greatness may not be overlooked he never fails to mention that Dr. Miller shook hands with him or stopped at his house, etc. I know most graduates are not capable of separating the college from the profession, and the bitter feeling and knowledge of being used to enrich the college stays with them when they start in practice.

Coagulation of the albumen in the tooth has furnished a favorite topic for some time. Having practiced chemistry for many years, naturally I use my chemical eye to view such matters. In considering the discoloration of a tooth by an amalgam filling containing copper, we are told that in proportion as it discolors it saves a tooth. Why? I never saw it stated. It is due, not exactly to coagulations, but to the formation of copper albuminate, (which is now an article of commerce), analogous to the conversion of hide into leather through tannic acid. May not a dilute solution of a zinc-salt do the same thing? I do not know but zinc is an element which forms many combinations with other bodies. In speaking of the comparative penetrating power of chemical agents through albumen, one thing is not to be lost sight of, namely a glass tubule is not a dental tubule. The first is not effected by chemical agents, the latter is. Lime and magnesia neutralize carbolic acid, and zinc chloride is precipitated by alkalies.—*Dental Digest.*

